# Psychometric properties of the 10-item Connor-Davidson Resilience Scale in Chinese military personnel

**DOI:** 10.3389/fpsyg.2023.1163382

**Published:** 2023-08-04

**Authors:** Zhihao Tu, Jingwen He, Ziying Wang, Mingfang Song, Jianquan Tian, Chuan Wang, Jianbo Ba, Xinghua Shen

**Affiliations:** ^1^Navy Special Medical Center, Naval Medical University, Shanghai, China; ^2^Qingdao Special Servicemen Recuperation Center of PLA Navy, Qingdao, Shandong, China; ^3^Department of Medical Psychology, No. 96110 Hospital, Yinchuan, Ningxia, China; ^4^Second Military Medical University, Shanghai, China

**Keywords:** resilience, 10-item Connor-Davidson Resilience Scale, psychometric, measurement invariance, military personnel

## Abstract

**Background:**

The 10-item Connor-Davidson Resilience Scale (CD-RISC-10) is a widely used assessment of resilience. However, psychometric properties of the Chinese version of CD-RISC-10 have not been well investigated in a Chinese military personnel sample.

**Methods:**

A total of 3,129 Chinese military personnel completed the CD-RISC-10, Self-rating Anxiety Scale (SAS), and Self-rating Depression Scale (SDS). Among them, 528 recruits completed the CD-RISC-10, SAS, and SDS again after 3-month basic military training (BMT). Meanwhile, the commanding officers were asked to rate recruits' training performance on the training performance rating scale for recruits (TPRS). Confirmatory factor analysis (CFA) was implemented to examine the single-factor model of the CD-RISC-10, and multigroup CFA was conducted to test measurement invariance across military rank (officers vs. enlisted), gender (male vs. female), and time (before and after 3-month BMT). Internal consistency was evaluated using Cronbach's α and McDonald's ω, and test–retest reliability was tested using the intra-class correlation coefficient (ICC). The criterion-related validity of CD-RISC was evaluated using Pearson's correlation analysis between the CD-RISC-10 total score and SAS scores, SDS scores, and training performance ratings.

**Results:**

The single-factor model of the CD-RISC-10 showed adequate fit (CFI = 0.955–0.970, TLI = 0.943–0.962, RMSEA = 0.059–0.072) in all examined subsamples (male, female, officer, and enlisted), and strict invariance was also supported across military rank, gender, and time (ΔCFI ≤ 0.001, ΔTLI ≤ 0.005, ΔRMSEA ≤ 0.006). The CD-RISC-10 showed good internal consistency in all subsamples (Cronbach's α of > 0.93 and McDonald's ω of > 0.93) and good test–retest reliability (ICC = 0.88). Moreover, concurrent and predictive validity with the SAS and SDS scores were good (*r* = −0.68 to −0.49, *p* < 0.001). The resilience level of recruits at the beginning of BMT was significantly associated with training performance rated by supervisors after training (*r* = 0.29, *p* < 0.001).

**Conclusion:**

The psychometric evidence reported in this study suggests that the CD-RISC-10 is a reliable and valid assessment of resilience and a potential predictor for mental health and military performance in Chinese military personnel.

## 1. Introduction

Resilience has been defined as the personal ability that enables one to overcome serious adversities (Connor and Davidson, 2003; Tugade and Fredrickson, 2004). Recently, researchers are inclined to regard resilience as a dynamic process, in which an individual takes full advantage of one's own internal and external resources to cope with stress and grow to adapt to future stressors (Richardson, 2002; Green et al., 2014; Cheng et al., 2020). Extensive research proved that resilience was a major protective factor in the face of challenges or stressors and that people with higher resilience were less vulnerable to prolonged psychological distress induced by negative events (Blanc et al., 2016; Wingo et al., 2017; Ran et al., 2020; Song et al., 2021). Moreover, growing evidence suggested that resilience might have a positive effect on job performance (Hou et al., 2020; Hosgor and Yaman, 2022; Kuşcu et al., 2022).

Military personnel are exposed to a highly stressful military environment throughout their enlistment, which makes them more susceptible to stress-related mental disorders, such as depression, anxiety, and posttraumatic stress disorder (PTSD) (Bezdjian et al., 2017). A recent large-scale epidemiological study reported that the prevalence rate of depression was 18.1% in Chinese military personnel (Feng et al., 2013). A meta-analysis showed that 12% of currently deployed U.S. military personnel met the criteria of major depression according to the Diagnostic and Statistical Manual of Mental Disorders-IV (DSM-IV) (Gadermann et al., 2012). More than 10% of soldiers returning from the Iraq War suffered from PTSD (Milliken et al., 2007). A high prevalence of mental illness among military personnel would significantly impair combat effectiveness and individual wellbeing (Pflanz and Ogle, 2006; Haran et al., 2019; Vogt et al., 2021; Bufford et al., 2022). In order to prevent and mitigate the adverse outcomes of military stress, many countries have been implementing resilience-oriented training programs and personnel selection aiming to maintain mental health and enhance performance in the military context (Cornum et al., 2011). For example, the U.S. Army implemented a universal program called Comprehensive Soldier Fitness (CSF) in order to develop psychological resilience in the army community (Casey, 2011; Reivich et al., 2011). Subsequent studies suggested that resilience training programs in CSF could alleviate participants' behavioral health symptoms (Griffith and West, 2013). In China, some researchers also developed a resilience training program and detected improvement in trainees' emotional state after training (Peng et al., 2014). In addition, previous studies also found that resilience had potential value as a predictor in military personnel selection. Oprins et al. (2021) found that resilience could predict military training performance in soldiers from European Defense Organizations. The predictive ability of resilience to training performance was also demonstrated within a sample of Israeli defense force combat officer candidates (Iversen et al., 2022).

Assessing resilience properly is first necessary for the research and application of resilience-oriented military personnel selection and training programs, which requires measurement tools with adequate evidence of reliability and validity (Xie et al., 2016; Bezdjian et al., 2017). Windle et al. (2011) reviewed 19 resilience measures and concluded that the Connor-Davidson Resilience Scale (CD-RISC) had the best psychometric properties. CD-RISC was a 25-item self-reported scale developed by Connor and Davidson (2003). This scale has been widely used and presented good psychometric properties in different countries and demographic groups (Bezdjian et al., 2017; Garcia-Leon et al., 2019; Velickovic et al., 2020; Dominguez-Cancino et al., 2022). However, the factor structure of CD-RISC lacked robust replication in different samples (Xie et al., 2016). For example, Yu and Zhang (2007) obtained a three-factor structure of CD-RISC in a general population sample, while Wu et al. (2017) obtained a four-factor structure of CD-RISC in a new employee population sample. In order to solve the problem of unstable factor structure, Campbell-Sills and Stein (2007) refined the original CD-RISC and established a 10-item version of CD-RISC (CD-RISC-10). The CD-RISC-10 presented a stable unidimensional structure across different populations (Coates et al., 2013; Cheng et al., 2020; Rezaeipandari et al., 2022). Furthermore, a considerable amount of literature suggested that the CD-RISC-10 had excellent internal consistency and criterion-related validity (Cheng et al., 2020). In view of superior psychometric properties, some researchers proposed that the CD-RISC-10 had higher priority than the original version of CD-RISC when considering resilience measures in studies (Campbell-Sills and Stein, 2007; Goins et al., 2013; Cheng et al., 2020).

Despite the promise of CD-RISC-10 as a valuable measurement tool for resilience, the reliability and validity, particularly, measurement invariance and predictive validity, of the Chinese version of CD-RISC-10 have not been well investigated in Chinese military personnel samples (Cheng et al., 2020). Measurement invariance is a necessary condition for valid group comparison and data pooling, which reflects that the scale used by researchers measures the same construct in an equivalent manner across different groups (Schmitt and Kuljanin, 2008; Gucciardi et al., 2011; Cheng et al., 2020). However, the measurement invariance of CD-RISC-10 was seldom examined in previous research, despite the fact that the resilience levels of different demographic groups were recorded and compared (Campbell-Sills and Stein, 2007; Stein et al., 2009; Gonzalez et al., 2016).

The army is a huge organization composed of highly heterogeneous populations, thus a test of measurement invariance of different populations in the army has both theoretical and practical values. For example, officers and soldiers follow completely independent processes of selection, training, and promotion, and they significantly differ in education, salary, and length of military service. Female officers and soldiers are relatively rare in the army, and most of them are assigned to less dangerous occupations. It is of great importance to compare group means of resilience and investigate the stability of its factor structure across gender and military rank in the army. In addition to the comparison of resilience levels between different demographic groups, researchers are also interested in investigating the changes in participants' resilience level before and after the resilience training program in order to examine the actual effect of the training program. For example, Peng et al. (2014) measured the resilience of Chinese military medical students using CD-RISC before and after the Penn Resilience Program (PRP) training and found that students obtained higher resilience scores after training. However, without any mention of evidence for longitudinal measurement invariance of CD-RISC, the authors claimed that the result demonstrated the positive effect of PRP on students' resilience level. In fact, evidence for longitudinal measurement invariance of a scale is indispensable to interpret the detected difference of scale scores across testing occasions as real changes induced by interventions (Bowers et al., 2010; Fried et al., 2016). However, up to now, there has been no detailed evidence for (longitudinal) measurement invariance of CD-RISC-10 in Chinese military personnel.

Although some studies supported the potential values of resilience as a predictor of military personnel selection because of its predictive validity for military performance, those studies used newly developed resilience scale, such as the INSPIRE resilience scale, rather than widely used CD-RISC or CD-RISC-10 (Oprins et al., 2021; Iversen et al., 2022). The details of those newly developed resilience scales (e.g., the INSPIRE resilience scale) are confidential, thus researchers from other countries cannot use those scales for further study. On the other hand, some studies demonstrated the predictive validity of CD-RISC or CD-RISC-10 for other military outcomes rather than military performance ratings, while performance ratings may be the “golden standard” criterion in the study of military personnel selection (Campbell et al., 1990; Mchenry et al., 1990). For example, Bezdjian et al. (2017) reported satisfactory predictive validity of the CD-RISC total score for unsuitability attrition and mental health diagnosis in a large United States Air Force to recruit sample (*N* = 53,692). In civilian settings, Hou et al. (2020) reported concurrent validity of the CD-RISC total score for job performance ratings among residents. Overall, few studies investigated the predictive validity of the CD-RISC-10 for military performance ratings.

The aim of the present study was to evaluate the psychometric properties of the CD-RISC-10 in Chinese military personnel. Specific goals were as follows: (1) confirming the single-factor structure of the CD-RISC-10; (2) examining measurement invariance across gender and military rank; (3) examining longitudinal measurement invariance before and after 3-month basic military training; (4) testing internal consistency reliability and test–retest reliability (3-month time interval); and (5) providing the evidence of concurrent and predictive validity with psychopathology measures and training performance ratings.

## 2. Materials and methods

### 2.1. Participants and procedure

Data were collected from 3,150 Chinese military personnel in Shandong Province and Hainan Province from 2021 to 2022. A total of 3,129 completed study questionnaires (response rate = 99.33%). The total sample contained 2,498 (79.83%) men and 859 (27.45%) officers. Among all the participants, 955 (30.52%) had higher education, and 2,025 (64.72%) were married. The average age of the participants, ranging from 18 to 48, was 26.50 (SD = 5.46) years.

All the participants were asked to complete the self-report questionnaires (CD-RISC-10, Zung Self-rating depression scale, and Zung Self-rating depression scale) in 1 h. Of the participants, 528 were recruits under basic military training when they first completed the questionnaires. After 3 months, these 528 recruits all finished the basic training program and were asked to fill in the self-report questionnaires again. At the same time, the commanding officers of these recruits' platoons were asked to rate the training performance of their own subordinates on the training performance rating scale for recruits (TPRS). All selected recruits were male and single in this study. The average age of the recruits was 19.58 (SD = 0.60) years. A total of 461 (87.31%) of the selected recruits were high school graduates.

This study received ethical approval from the Committee on Second Military Medical University. The study was conducted in accordance with the Helsinki guidelines. All participants signed written informed consent before completing the study questionnaires.

### 2.2. Measures

#### 2.2.1. The CD-RISC-10

The Chinese version of CD-RISC-10 was used to assess resilience in this study (Wang et al., 2010). The CD-RISC-10 consists of 10 questions, and the responses are on a 5-point Likert scale ranging from 0 (“not true at all”) to 4 (“true nearly all of the time”), generating total scores from 0 to 40 (Campbell-Sills and Stein, 2007). The higher total scores indicate a greater ability to cope with adversity (Cheng et al., 2020). The psychometric properties of CD-RISC-10 were well developed in a Chinese undergraduate sample (Cheng et al., 2020).

#### 2.2.2. The Zung Self-rating anxiety scale

The Self-rating anxiety scale (SAS) consists of 20 items and was developed to measure the frequency of anxiety symptoms in the last week (Zung, 1971). The item responses are on a 4-point Likert scale ranging from 1 (“none or a little of the time”) to 4 (“most or all of the time”), generating total scores from 20 to 80. The Chinese version of SAS was used in this study (Liu et al., 1997). The reliability and validity of SAS were demonstrated in Chinese samples (Gao et al., 2012; Gong et al., 2014). In this study, the internal reliability of SAS was acceptable: Cronbach's α = 0.86, McDonald's ω = 0.84.

#### 2.2.3. The Zung Self-rating depression scale

The Self-rating depression scale (SDS) consists of 20 items and was developed to measure the frequency of depression symptoms in the last week (Zung, 1965). The item responses are on a 4-point Likert scale ranging from 1 (“none or a little of the time”) to 4 (“most or all of the time”), generating total scores from 20 to 80. The Chinese version of SDS was used in this study (Liu et al., 1999). The reliability and validity of SDS were demonstrated in Chinese samples (Lee et al., 1994; Liu et al., 1999). In this study, the internal reliability of SDS was acceptable: Cronbach's α = 0.88, McDonald's ω = 0.89.

#### 2.2.4. Training performance rating scale for recruits

Training performance rating scale for recruits (TPRS) was a 12-item supervisor-rating scale developed by Tu et al. (2020) to measure recruits' performance during the 3-month basic military training program. The item responses are on a 7-point Likert scale ranging from 1 (“extremely bad”) to 7 (“extremely good”), generating total scores from 12 to 84. The higher total scores indicate better training performance. The commanding officer of the participant's platoon was asked to rate the participant's training performance on TPRS. In this study, the internal reliability of TPRS was acceptable: Cronbach's α = 0.95, McDonald's ω = 0.95.

### 2.3. Statistical analysis

Analyses were performed using R software (version 4.2.2) for Windows (R Core Team, 2022). To study item characteristics, we calculated the means, standard deviation (SD), skewness, kurtosis, corrected item-total score correlations, Cronbach's α, and α-if-item-deleted (Cheng et al., 2020). According to George and Mallery (2010), skewness and kurtosis values between −2 and +2 are regarded as acceptable for demonstrating a normal univariate distribution. The values of corrected item-total score correlations are acceptable between 0.40 and 0.80, which indicates that all items test the same underlying psychological construct without multicollinearity (Cheng et al., 2020). The independent-sample *t*-test was used to compare mean differences in total CD-RISC-10 scores between the officer and enlisted groups as well as male and female groups, while the paired-sample *t*-test was used to compare mean differences in total CD-RISC-10 scores between the test and retest samples. The effect size of mean differences was assessed by Cohen's *d* (Cohen, 1988).

Confirmatory factor analyses (CFA) were performed using R package lavaan (Rosseel, 2012) to examine the unidimensional structure of CD-RISC-10 in different gender, military rank, or test–retest groups independently. The robust maximum likelihood estimator (MLR) was selected for model estimation (Cheng et al., 2020). Model fit was accessed by the Tucker–Lewis index (TLI), comparative fit index (CFI), and root mean square error of approximation (RMSEA) (Marsh et al., 2004). According to Hu and Bentler (1998), TLI ≥ 0.90, CFI ≥ 0.90, and RMSEA ≤ 0.08 were considered to indicate an acceptable model fit.

Measurement invariance of CD-RISC-10 across different groups (gender and military rank) was examined using a multigroup confirmatory factor analysis (MG-CFA). The measurement invariance test procedures were as follows (Millsap and Yun-Tein, 2004): first, configural invariance was evaluated to check whether the groups share the same general factorial pattern of the measure. In this sector, all parameters were freely estimated. Second, weak invariance was assessed to examine whether the groups display the same factor loadings. In this sector, the factor loadings were constrained to be equivalent between groups. Third, strong invariance was accessed to check whether a unit variation at the level of the latent variable is linked to an identical change at the level of the scores for items in the groups. In this sector, the factor loadings and items' intercepts were constrained to be equivalent between groups. Fourth, strict invariance was evaluated to examine whether measurement error variance was also equivalent between groups. In this sector, the factor loadings, items' intercepts, and measurement error variance were all constrained to be equivalent between groups. According to Chen (2007), when the sample size is adequate (total *N* > 300), the following cutoff points for testing invariance are appropriate: ΔCFI < 0.010, ΔTLI < 0.010, ΔRMSEA < 0.015. The procedure of evaluation for longitudinal measurement invariance was basically consistent with that of cross-sectional measurement invariance described above, except that the common factors and the unique factors for each indicator were allowed to correlate over two test occasions (Widaman et al., 2010; Liu et al., 2017).

In addition, the R package psych (Revelle, 2022) was used to assess the internal consistency reliability. The internal consistency reliability of the CD-RISC-10 was examined using Cronbach's α and McDonald's ω (Revelle and Zinbarg, 2009). Cronbach's α of ≥ 0.70 and McDonald's ω of ≥0.80 are considered to indicate good internal consistency reliability (Cohen et al., 2007; Feisst et al., 2019). The test–retest reliability of the CD-RISC-10 over the two test occasions was analyzed using a one-way random effects model intraclass correlation coefficient (ICC) (Kernot et al., 2015) by R package lmerTest (Kuznetsova et al., 2017). According to Koo and Li (2016), 0.50 ≤ ICC < 0.75, 0.75 ≤ ICC < 0.90, and ICC ≥ 0.90 indicate moderate, good, and excellent test–retest reliability, respectively. The criterion-related validity of CD-RISC was evaluated by Pearson's correlation analysis between the CD-RISC-10 total score and different criteria (SAS scores, SDS scores, and training performance ratings).

## 3. Results

### 3.1. Missing data

Missing responses for the CD-RISC-10 items were 0.003% in the whole sample. The amount of missing data was not substantial, and the pattern of missing data was shown to be random (Penelo et al., 2017; Cheng et al., 2020), thus missing data were treated using the full information maximum likelihood method in the present study (Schafer and Graham, 2002).

### 3.2. Item characteristics and descriptive statistics

The item characteristics and descriptive statistics of CD-RISC-10 are presented in Tables 1–3 for the officer and enlisted groups, the male and female groups, and the test and retest recruits samples, respectively. The skewness and kurtosis values of these samples were between −2 and +2, which suggested all items of CD-RISC-10 could be regarded as acceptable as normal univariate distributions (George and Mallery, 2010; Cheng et al., 2020). In addition, the corrected item-total score correlations, ranging from 0.61 (Item 1 in the pre-training recruit sample) to 0.80 (Item 4 in the pre-training recruit sample), were all between 0.40 and 0.80, which indicated that all items tested the same underlying psychological construct without multicollinearity (Cheng et al., 2020).

**Table 1:** Mean, SDs, skewness, kurtosis, Cronbach's α and α-if-item-deleted (α), corrected item-total score correlations (*r*_tt_), and factor loadings (Loading) of the CD-RISC-10 in the officer and enlisted groups.

**Item**	**Mean ±SD**	**Skewness**	**Kurtosis**	**α**	***r*_tt_**	**Loading**
**Officer group (*****N*** = **859)**						
1. Able to adapt to change	3.42 ± 0.81	−1.52	1.98	0.94	0.65	0.54
2. Can deal with whatever comes	2.96 ± 0.94	−0.78	0.47	0.94	0.72	0.69
3. Tries to see humorous side of problems	3.01 ± 0.97	−0.82	0.31	0.94	0.72	0.72
4. Coping with stress can strengthen me	2.89 ± 1.02	−0.67	−0.19	0.93	0.79	0.85
5. Tend to bounce back after illness or hardship	3.08 ± 0.96	−0.96	0.52	0.94	0.77	0.76
6. Can achieve goals despite obstacles	3.20 ± 0.89	−0.88	0.13	0.94	0.75	0.69
7. Can stay focused under pressure	3.10 ± 0.94	−0.86	0.16	0.94	0.78	0.76
8. Not easily discouraged by failure	3.22 ± 0.95	−1.18	1.01	0.93	0.80	0.80
9. Thinks of self as strong person	3.08 ± 1.01	−1.03	0.65	0.93	0.80	0.87
10. Can handle unpleasant feelings	3.14 ± 0.95	−0.93	0.24	0.94	0.78	0.77
Total score	31.11 ± 7.66	−0.94	0.85	0.94		
**Enlisted group (*****N*** = **2,270)**						
1. Able to adapt to change	3.40 ± 0.83	−1.42	1.83	0.94	0.65	0.56
2. Can deal with whatever comes	2.97 ± 0.89	−0.64	0.18	0.93	0.71	0.65
3. Tries to see humorous side of problems	3.01 ± 0.95	−0.70	−0.09	0.94	0.67	0.66
4. Coping with stress can strengthen me	2.89 ± 1.00	−0.62	−0.20	0.93	0.80	0.82
5. Tend to bounce back after illness or hardship	3.08 ± 0.92	−0.85	0.35	0.93	0.78	0.74
6. Can achieve goals despite obstacles	3.20 ± 0.88	−0.87	0.16	0.93	0.75	0.68
7. Can stay focused under pressure	3.05 ± 0.94	−0.74	0.00	0.93	0.79	0.78
8. Not easily discouraged by failure	3.20 ± 0.92	−1.07	0.82	0.93	0.78	0.74
9. Thinks of self as strong person	3.09 ± 0.97	−0.90	0.33	0.93	0.79	0.80
10. Can handle unpleasant feelings	3.14 ± 0.92	−0.87	0.18	0.93	0.77	0.73
Total score	31.03 ± 7.39	−0.83	0.57	0.94		

SD, standard deviation; CD-RISC-10, 10-item Connor–Davidson Resilience Scale.

**Table 2:** Mean, SDs, skewness, kurtosis, Cronbach's α and α-if-item-deleted (α), corrected item-total score correlations (*r*_tt_), and factor loadings (Loading) of the CD-RISC-10 in the male and female groups.

**Item**	**Mean ±SD**	**Skewness**	**Kurtosis**	**α**	***r*_tt_**	**Loading**
**Male group (*****N*** = **2,498)**						
1. Able to adapt to change	3.40 ± 0.83	−1.44	1.89	0.94	0.65	0.56
2. Can deal with whatever comes	2.97 ± 0.90	−0.64	0.13	0.93	0.71	0.66
3. Tries to see humorous side of problems	3.01 ± 0.95	−0.70	−0.05	0.94	0.69	0.67
4. Coping with stress can strengthen me	2.89 ± 1.00	−0.62	−0.22	0.93	0.80	0.83
5. Tend to bounce back after illness or hardship	3.07 ± 0.93	−0.86	0.34	0.93	0.78	0.75
6. Can achieve goals despite obstacles	3.21 ± 0.88	−0.87	0.09	0.93	0.74	0.68
7. Can stay focused under pressure	3.05 ± 0.94	−0.74	−0.04	0.93	0.79	0.77
8. Not easily discouraged by failure	3.19 ± 0.93	−1.08	0.79	0.93	0.79	0.77
9. Thinks of self as strong person	3.09 ± 0.98	−0.93	0.40	0.93	0.80	0.82
10. Can handle unpleasant feelings	3.14 ± 0.93	−0.87	0.09	0.93	0.77	0.74
Total score	31.02 ± 7.47	−0.81	0.48	0.94		
**Female group (*****N*** = **631)**						
1. Able to adapt to change	3.43 ± 0.80	−1.48	1.36	0.94	0.67	0.54
2. Can deal with whatever comes	2.96 ± 0.91	−0.87	0.85	0.94	0.71	0.66
3. Tries to see humorous side of problems	3.04 ± 0.97	−0.87	0.33	0.94	0.69	0.68
4. Coping with stress can strengthen me	2.90 ± 1.02	−0.71	−0.11	0.93	0.79	0.82
5. Tend to bounce back after illness or hardship	3.10 ± 0.92	−0.97	0.69	0.93	0.78	0.73
6. Can achieve goals despite obstacles	3.17 ± 0.89	−0.90	0.37	0.93	0.77	0.71
7. Can stay focused under pressure	3.10 ± 0.92	−0.90	0.41	0.93	0.79	0.76
8. Not easily discouraged by failure	3.25 ± 0.90	−1.19	1.23	0.93	0.77	0.73
9. Thinks of self as strong person	3.09 ± 0.98	−0.97	0.62	0.93	0.80	0.82
10. Can handle unpleasant feelings	3.13 ± 0.91	−0.98	0.66	0.93	0.79	0.75
Total score	31.17 ± 7.43	−1.04	1.38	0.94		

SD, standard deviation; CD-RISC-10, 10-item Connor–Davidson Resilience Scale.

**Table 3:** Mean, SDs, skewness, kurtosis, Cronbach's α and α-if-item-deleted (α), corrected item-total score correlations (*r*_tt_), and factor loadings (Loading) of the CD-RISC-10 in the pre- and post-training recruit groups.

**Item**	**Mean ±SD**	**Skewness**	**Kurtosis**	**α**	***r*_tt_**	**Loading**
**Pre-training recruit group (*****N*** = **528)**						
1. Able to adapt to change	3.35 ± 0.84	−1.28	1.38	0.94	0.61	0.52
2. Can deal with whatever comes	2.90 ± 0.89	−0.72	0.59	0.93	0.75	0.68
3. Tries to see humorous side of problems	2.96 ± 0.97	−0.73	0.13	0.93	0.67	0.66
4. Coping with stress can strengthen me	2.87 ± 1.01	−0.75	0.22	0.93	0.80	0.83
5. Tend to bounce back after illness or hardship	3.06 ± 0.91	−0.85	0.45	0.93	0.78	0.74
6. Can achieve goals despite obstacles	3.17 ± 0.87	−0.73	−0.24	0.93	0.73	0.66
7. Can stay focused under pressure	3.02 ± 0.94	−0.69	−0.09	0.93	0.76	0.75
8. Not easily discouraged by failure	3.16 ± 0.91	−0.99	0.59	0.93	0.79	0.76
9. Thinks of self as strong person	3.08 ± 0.98	−0.90	0.32	0.93	0.80	0.81
10. Can handle unpleasant feelings	3.07 ± 0.96	−0.84	0.21	0.93	0.78	0.77
Total score	30.65 ± 7.42	−0.88	0.69	0.94		
**Post-training recruit group (*****N*** = **528)**						
1. Able to adapt to change	3.39 ± 0.85	−1.52	1.23	0.94	0.66	0.58
2. Can deal with whatever comes	2.99 ± 0.87	−0.64	0.14	0.93	0.73	0.65
3. Tries to see humorous side of problems	3.03 ± 0.93	−0.70	−0.06	0.94	0.64	0.61
4. Coping with stress can strengthen me	2.91 ± 0.97	−0.53	−0.47	0.93	0.80	0.80
5. Tend to bounce back after illness or hardship	3.09 ± 0.90	−0.92	0.59	0.93	0.80	0.74
6. Can achieve goals despite obstacles	3.25 ± 0.85	−0.92	0.35	0.93	0.75	0.66
7. Can stay focused under pressure	3.08 ± 0.92	−0.83	0.31	0.93	0.80	0.77
8. Not easily discouraged by failure	3.22 ± 0.91	−1.16	1.19	0.93	0.79	0.75
9. Thinks of self as strong person	3.10 ± 0.94	−0.82	0.13	0.93	0.78	0.76
10. Can handle unpleasant feelings	3.19 ± 0.85	−0.95	0.66	0.93	0.78	0.68
Total score	31.26 ± 7.22	−0.85	0.72	0.94		

SD, standard deviation; CD-RISC-10, 10-item Connor–Davidson Resilience Scale.

Total scores of CD-RISC-10 ranged from 0 to 40 (Mean = 31.11; SD = 7.66) in the officer group, while the enlisted group ranged from 0 to 40 (Mean = 31.03; SD = 7.39). The independent-sample *t*-test analysis showed that total CD-RISC-10 scores were not significantly different between the officer and enlisted groups (*t* = 0.27, *p* = 0.789, Cohen's *d* = 0.01). Total scores of CD-RISC-10 ranged from 0 to 40 (Mean = 31.02; SD = 7.47) in the male group, while the female group ranged from 0 to 40 (Mean = 31.17; SD = 7.43). The independent-sample *t*-test analysis showed that total CD-RISC-10 scores were not significantly different between the male and female groups (*t* = −0.45, *p* = 0.656, Cohen's *d* = −0.02). Total scores of CD-RISC-10 ranged from 0 to 40 (Mean = 30.65; SD = 7.42) in the pre-training recruits group, while the post-training recruits group ranged from 0 to 40 (Mean = 31.26; SD = 7.22). The paired independent-sample *t*-test analysis showed that total CD-RISC-10 scores were significantly higher in recruits before basic military training than that after training (*t* = −4.01, *p* < 0.001, Cohen's *d* = −0.17).

### 3.3. Structural validity

All item loadings, ranging from 0.52 to 0.87, significantly loaded on the latent factor (*p* < 0.001) in each examined sample (see Tables 1–3). The results of the single-factor confirmatory factor analysis in each examined sample are presented in Table 4. The results showed that the model fit of the single-factor model was acceptable for all the groups (officer group: CFI = 0.970, TLI = 0.961, RMSEA = 0.059; enlisted group: CFI = 0.966, TLI = 0.957, RMSEA = 0.063; male group: CFI = 0.970, TLI = 0.962, RMSEA = 0.059; female group: CFI = 0.955, TLI = 0.943, RMSEA = 0.072; pre-training recruit group: CFI = 0.963, TLI = 0.953, RMSEA = 0.065; and post-training recruit group: CFI = 0.959, TLI = 0.948, RMSEA = 0.066). These results suggested that the unidimensional structure of the CD-RISC-10 was stable among the officer and enlisted groups, the male and female groups, and the test and retest samples.

**Table 4:** Model fit of single-factor confirmatory factor analysis (CFA) in the officer and enlisted groups, the male and female groups, and the pre- and post-training recruit groups.

**Model**	**χ2**	***df***	**CFI**	**TLI**	**RMSEA (90% CIs)**
Officer	223.226^***^	35	0.970	0.961	0.059 (0.051–0.067)
Enlisted	563.139^***^	35	0.966	0.957	0.063 (0.058–0.067)
Male	553.672^***^	35	0.970	0.962	0.059 (0.054–0.063)
Female	234.529^***^	35	0.955	0.943	0.072 (0.063–0.082)
Pre-training recruit	167.631^***^	35	0.963	0.953	0.065 (0.055–0.077)
Post-training recruit	185.182^***^	35	0.959	0.948	0.066 (0.056–0.077)

χ^2^, chi-square goodness of fit; df, degrees of freedom; CFI, comparative fit index; TLI, Tucker–Lewis index; RMSEA, root mean square error of approximation; 90% Cis, 90% confidence intervals for RMSEA.

^*^p < 0.05.

^**^p < 0.01.

^***^p < 0.001.

### 3.4. Measurement invariance

#### 3.4.1. Officer group vs. enlisted group

The results of multigroup CFA were as follows: configural invariance model (CFI = 0.967, TLI = 0.958, RMSEA = 0.062), weak invariance model (ΔCFI = 0.000, ΔTLI = 0.004, ΔRMSEA = −0.003), strong invariance model (ΔCFI = 0.000, ΔTLI = 0.004, ΔRMSEA = −0.002), and strict invariance model (ΔCFI = 0.000, ΔTLI = 0.004, ΔRMSEA = −0.005). These results supported the measurement invariance of the CD-RISC-10 across the officer and enlisted groups (see Table 5).

**Table 5:** Measurement invariance testing results of the CD-RISC-10 across military rank.

**Model**	**χ^2^**	***df***	**CFI**	**TLI**	**RMSEA (90%CIs)**	**Comparison**	**ΔCFI**	**ΔTLI**	**ΔRMSEA**
1. Configural	786.365^***^	70	0.967	0.958	0.062 (0.058–0.066)				
2. Weak	799.350^***^	79	0.967	0.962	0.059 (0.055–0.063)	2 vs. 1	0.000	0.004	−0.003
3. Strong	805.529^***^	88	0.967	0.966	0.057 (0.053–0.061)	3 vs. 2	0.000	0.004	−0.002
4. Strict	820.725^***^	98	0.967	0.970	0.052 (0.049–0.056)	4 vs. 3	0.000	0.004	−0.005

χ^2^, chi-square goodness of fit; df, degrees of freedom; CFI, comparative fit index; TLI, Tucker–Lewis index; RMSEA, root mean square error of approximation; 90% Cis, 90% confidence intervals for RMSEA; ΔCFI, CFI difference; ΔTLI, TLI difference; ΔRMSEA, RMSEA difference.

^*^p < 0.05.

^**^p < 0.01.

^***^p < 0.001.

#### 3.4.2. Male group vs. female group

The results of multigroup CFA were as follows: configural invariance model (CFI = 0.967, TLI = 0.958, RMSEA = 0.062), weak invariance model (ΔCFI = 0.000, ΔTLI = 0.005, ΔRMSEA = −0.003), strong invariance model (ΔCFI = 0.000, ΔTLI = 0.003, ΔRMSEA = −0.002), and strict invariance model (ΔCFI = 0.000, ΔTLI = 0.004, ΔRMSEA = −0.004). These results supported the measurement invariance of the CD-RISC-10 across the male and female groups (see Table 6).

**Table 6:** Measurement invariance testing results of the CD-RISC-10 across gender.

**Model**	**χ2**	***df***	**CFI**	**TLI**	**RMSEA (90%CIs)**	**Comparison**	**ΔCFI**	**ΔTLI**	**ΔRMSEA**
1. Configural	788.202^***^	70	0.967	0.958	0.062 (0.058–0.066)				
2. Weak	793.728^***^	79	0.967	0.963	0.059 (0.055–0.063)	2 vs. 1	0.000	0.005	−0.003
3. Strong	806.182^***^	88	0.967	0.966	0.057 (0.053–0.061)	3 vs. 2	0.000	0.003	−0.002
4. Strict	817.195^***^	98	0.967	0.970	0.053 (0.049–0.056)	4 vs. 3	0.000	0.004	−0.004

χ^2^, chi-square goodness of fit; df, degrees of freedom; CFI, comparative fit index; TLI, Tucker–Lewis index; RMSEA, root mean square error of approximation; 90% Cis, 90% confidence intervals for RMSEA; ΔCFI, CFI difference; ΔTLI, TLI difference; ΔRMSEA, RMSEA difference.

^*^p < 0.05.

^**^p < 0.01.

^***^p < 0.001.

#### 3.4.3. Pre-training recruit group vs. post-training recruit group

The results of multigroup CFA were as follows: configural invariance model (CFI = 0.961, TLI = 0.950, RMSEA = 0.062), weak invariance model (ΔCFI = 0.000, ΔTLI = 0.005, ΔRMSEA = −0.003), strong invariance model (ΔCFI = 0.000, ΔTLI = 0.003, ΔRMSEA = −0.002), and strict invariance model (ΔCFI = 0.000, ΔTLI = 0.004, ΔRMSEA = −0.004). These results supported the longitudinal measurement invariance of the CD-RISC-10 (see Table 7).

**Table 7:** Measurement invariance testing results of the CD-RISC-10 across gender.

**Model**	**χ2**	***df***	**CFI**	**TLI**	**RMSEA (90%CIs)**	**Comparison**	**ΔCFI**	**ΔTLI**	**ΔRMSEA**
1. Configural	352.813^***^	60	0.961	0.950	0.066 (0.058–0.074)				
2. Weak	364.264^***^	69	0.961	0.955	0.063 (0.056–0.071)	2 vs. 1	0.000	0.005	−0.003
3. Strong	372.296^***^	78	0.961	0.960	0.061 (0.054–0.068)	3 vs. 2	0.000	0.005	−0.002
4. Strict	389.191^***^	88	0.960	0.963	0.055 (0.049–0.062)	4 vs. 3	−0.001	0.003	−0.006

χ^2^, chi-square goodness of fit; df, degrees of freedom; CFI, comparative fit index; TLI, Tucker–Lewis index; RMSEA, root mean square error of approximation; 90% CIs, 90% confidence intervals for RMSEA; ΔCFI, CFI difference; ΔTLI, TLI difference; ΔRMSEA, RMSEA difference.

^*^p < 0.05.

^**^p < 0.01.

^***^p < 0.001.

### 3.5. Test–retest reliability

The CD-RISC-10 total scores of tested recruits were highly correlated between the test and retest (*r* = 0.89, *t* = 43.86, *p* < 0.001). The results of the one-way random effects model showed that ICC = 0.88, which indicated good test–retest reliability of the CD-RISC-10.

### 3.6. Internal consistency reliability

In the whole sample and each examined group, all Cronbach's α coefficients were > 0.93 and McDonald's ω was > 0.93 (see Table 8), which suggested that the CD-RISC-10 had satisfactory internal consistency reliability.

**Table 8:** The internal consistency reliability of CD-RISC-10 in the whole sample, officer and Enlisted groups, the male and female groups, and the pre- and post-training recruit groups.

**Sample**	**α (95% CIs)**	**ω (95% CIs)**
Whole sample (*n* = 3,129)	0.94 (0.94–0.94)	0.94 (0.94–0.94)
Officer group (*n* = 859)	0.94 (0.94–0.95)	0.94 (0.94–0.95)
Enlisted group (*n* = 2,270)	0.94 (0.93–0.94)	0.94 (0.93–0.94)
Male group (*n* = 2,498)	0.94 (0.94–0.94)	0.94 (0.94–0.94)
Female group (*n* = 631)	0.94 (0.93–0.95)	0.94 (0.93–0.95)
Pre-training recruit group (*n* = 528)	0.94 (0.93–0.94)	0.94 (0.93–0.95)
Post-training recruit group (*n* = 528)	0.94 (0.93–0.95)	0.94 (0.93–0.94)

α, Cronbach's α; ω, McDonald's ω; 95% CIs, 95% confidence intervals.

### 3.7. Criterion-related validity

Regarding the criterion-related validity, there was a strong negative correlation (*r* = −0.68 to −0.50, *p* < 0.001) between CD-RISC-10 and SAS and SDS in the whole sample, officer and enlisted groups, male and female groups, and pre-training recruit group (see Table 9), which indicated that the CD-RISC-10 had satisfactory concurrent validity for depression and anxiety symptoms. The CD-RISC-10 total scores before basic military training were positively associated with training performance rated by supervisors after training (*r* = 0.29, *t* = 6.96, *p* < 0.001), which suggested that the CD-RISC-10 had predictive value for military training performance. The CD-RISC-10 total scores before basic military training were negatively associated with depression (*r* = −0.53, *t* = 10.78, *p* < 0.001) and anxiety symptoms (*r* = −0.49, *t* = 12.94, *p* < 0.001) after training, which suggested that the CD-RISC-10 had predictive value for mental health.

**Table 9:** Correlation results of the CD-RISC-10 and the SAS, SDS, and TPRS.

**Sample**	**SAS**	**SDS**	**TPRS**
Whole sample (*n* = 3,129)	−0.57^***^	−0.65^***^	
Officer group (*n* = 859)	−0.50^***^	−0.61^***^	
Enlisted group (*n* = 2,270)	−0.59^***^	−0.66^***^	
Male group (*n* = 2,498)	−0.57^***^	−0.65^***^	
Female group (*n* = 631)	−0.55^***^	−0.65^***^	
Pre-training recruit group (*n* = 528)	−0.61^***^	−0.68^***^	
Post-training recruit group (*n* = 528)	−0.49^***^	−0.53^***^	0.29^***^

SAS, Zung Self-rating anxiety scale; SDS, Zung Self-rating depression scale; TPRS, Training performance rating scale for recruits; r, Pearson's correlation coefficient.

^*^p < 0.05.

^**^p < 0.01.

^***^p < 0.001.

## 4. Discussion

In the present study, we examined the psychometric properties of CD-RISC-10 in a Chinese military personnel sample, especially measurement invariance across different demographic groups (officer vs. enlisted; male vs. female) and longitudinal measurement invariance across time (before and after 3-month basic military training). To the best of our knowledge, this was the first study to evaluate measurement invariance (longitudinal and cross-sectional), test–retest reliability, and criterion-related validity (concurrent and predictive validity) of the CD-RISC-10 in a large-scale military sample. The results supported the expected unidimensional structure of the CD-RISC-10 and demonstrated strict invariance across military rank, gender, and time. The results also suggested that the CD-RISC-10 had good internal consistency and test–retest reliability, as well as concurrent and predictive validity in the military sample.

The unidimensional structure of CD-RISC-10 was supported by CFA results in this study, which accorded with earlier studies conducted in clinical or non-clinical, Eastern or Western culture, younger or older, and different occupational samples (Gucciardi et al., 2011; Blanco et al., 2019; Cheng et al., 2020; Rezaeipandari et al., 2022). This result suggested that the unidimensional structure of CD-RISC-10 was stable in different populations.

The results of the measurement invariance examination suggested that the CD-RISC-10 showed configural, weak, strong, and strict invariance across military rank, gender, and time. The measurement invariance of the CD-RISC-10 across gender was consistent with previous research (Gonzalez et al., 2016; Cheng et al., 2020). The present study was also in agreement with Chen et al. (2022), which also supported longitudinal measurement invariance of the CD-RISC-10 over a 6-month time interval in a Chinese children sample. Therefore, the present study suggested that the CD-RISC-10 held the same meaning and manner when measuring resilience across military rank, gender, and time in military personnel (Chen et al., 2022). In addition, given this evidence of measurement invariance, it is reasonable to directly compare the CD-RISC-10 total scores across time and different groups, and inter-group differences in scale scores were the actual differences in resilience levels (Cheng et al., 2020).

The present study found that there were no statistically significant differences in CD-RISC-10 total scores between the male and female groups. However, previous research found that male participants reported higher CD-RISC-10 scores than female participants in a Chinese undergraduate sample (Cheng et al., 2020). A possible explanation for this inconsistency might be that military personnel were all highly selective populations which can eliminate gender differences in resilience level. In addition, the comparison of CD-RISC-10 total scores measured before and after basic military training showed that recruits obtained higher scores post-training than they obtained pre-training. However, the effect size was really small (Cohen's *d* = −0.17), thus caution must be applied, as this finding might not be interpreted as the existence of a positive training effect on recruits' resilience level.

The results of reliability analysis showed that all Cronbach's α values were > 0.93 and McDonald's ω was > 0.93 in each examined subsample, which suggested that the CD-RISC-10 had satisfactory internal consistency reliability. This result was in accord with former research (Campbell-Sills and Stein, 2007; Cheng et al., 2020; Chen et al., 2022). In addition, the present study demonstrated that the CD-RISC-10 had good test–retest reliability (ICC = 0.88) in a recruit sample over 3 months. The test–retest reliability of the CD-RISC-10 in this study was slightly higher than that of previous studies, such as 0.665 over 6 months in a Chinese older adult sample (Meng et al., 2019). This result may be explained by the different time frames between test and retest occasions in these studies (3 months vs. 6 months). The satisfactory test–retest reliability confirmed that the construct measured by the CD-RISC-10 was relatively stable over time (Campbell-Sills and Stein, 2007; Notario-Pacheco et al., 2011; Tourunen et al., 2021).

With reference to the criterion-related validity, there were strong negative correlations between the CD-RISC-10 total scores and SAS and SDS total scores, which indicated that the resilience level of military personnel was associated with depression and anxiety symptoms. Previous studies also found that the resilience level of military personnel was related to anxiety and depression (Yu et al., 2016; van der Meulen et al., 2020; Guo et al., 2021). The relationship between resilience and depression could be attributed to genetic factors, neurobiological factors, and social factors (Southwick et al., 2005; Silk et al., 2007; Navrady et al., 2018). This study also found that there was a significant positive correlation between pre-training CD-RISC-10 total scores and post-training TPRS total scores in recruits, which indicated that recruits with higher resilience level were more likely to perform better in their early military career. Previous researchers paid more attention to the predictive values of psychological resilience on mental health in military personnel (Bezdjian et al., 2017; van der Meulen et al., 2020). As far as we know, the present study was the first attempt to report the predictive validity result of the CD-RISC-10 by using military training performance as a criterion. Although the correlation coefficient was relatively small (*r* = 0.29), the CD-RISC-10 is still a potential predictor for military personnel selection and is worth studying in the future (Oprins et al., 2021; Iversen et al., 2022).

## 5. Conclusion

In conclusion, the present study demonstrated that the CD-RISC-10 was a reliable and valid measure of resilience in military personnel. The results supported measurement invariance of the CD-RISC-10 across military rank, gender, and time. In addition, this study found that the CD-RISC-10 total score was strongly related to depression and anxiety symptoms, and it was predictive of military training performance. Given these findings, efforts to enhance resilience among military personnel may improve their mental health, and assessing the resilience of recruited candidates may be a useful procedure in military selection.

## Data availability statement

The raw data supporting the conclusions of this article will be made available by the authors, without undue reservation.

## Ethics statement

The studies involving human participants were reviewed and approved by Committee on Second Military Medical University. The patients/participants provided their written informed consent to participate in this study.

## Author contributions

ZT, JH, and ZW contributed to the study design, data input and analyses, drafting, and manuscript revision. JT, MS, and CW contributed to the questionnaire survey. XS and JB contributed to the manuscript revision. All authors have read and approved the final manuscript.
